# Breast Edema After Breast-Conserving Surgery and Radiotherapy: Introduction of a Clinically Meaningful Classification and Evaluation of the Incidence After Normo- and Hypofractionated Treatments

**DOI:** 10.3390/cancers17142368

**Published:** 2025-07-16

**Authors:** Melsa Rojin Oyur, Robert Maximilian Blach, Hans Christiansen, Roland Merten, Jan-Niklas Becker, Anne Caroline Knöchelmann, Mirko Nitsche, Robert Michael Hermann, Mathias Alexander Sonnhoff

**Affiliations:** 1 Hannover Medical School, Department of Radiotherapy, Carl-Neuberg-Straße 1, 30625 Hannover, Germany; 2 Radiotherapy North, Center for Radiotherapy and Radiooncology Westerstede, An der Hössen 34, 26655 Westerstede, Germany; 3 University Medical Center Schleswig-Holstein, Department of Radiation Oncology, 24105 Kiel, Germany; 4 IMAGINE-Niedersachsen, Carl-Neuberg-Str. 1, 30625 Hannover, Germany

**Keywords:** breast edema, radiotherapy side effects, breast-conserving therapy, classification of side effects in radiotherapy

## Abstract

BE following RT after breast-conserving surgery is a common concern, impacting patients’ quality of life. This study introduces a clinically meaningful classification system for BE and retrospectively evaluates its incidence among patients treated with normofractionated (nfRT) and hypofractionated (hfRT) regimens. Data from 1156 patients treated between 2011 and 2021 were analyzed. BE was graded according to the CTC and a so-called “WST classification” (grade 1: lymphatic drainage performed by the patient; grade 2: professional lymphatic drainage; grade 3: surgery). A total of 33%/17% developed BE according to the WST classification/CTC. Grade III BE was not reported. About 70% experienced a remission of BE during follow-up. Risk factors for the development of BE included RT of lymphatic drainage, complete axillary dissection compared to sentinel node dissection, and CTX. CTX was not confirmed in multivariate analysis. The incidence of BE did not differ significantly between the nfRT and hfRT groups, affirming the safety and comparability of hfRT regarding BE risk (HR: 0.833, *p* = 0.1219). This study emphasizes the importance of precise and standardized BE classification for improved treatment outcomes. Given its comparable risk profile and potential for enhanced therapy adherence, the findings support hfRT as a preferred regimen aligned with the current guidelines.

## Simple Summary

This study addresses a gap in the clinical classification of radiogenic breast edema (BE) in organ-conserving therapy for breast cancer. The classification focuses on the clinical consequences of treating BE. In addition, risk factors such as smoking, surgical procedures, and radiotherapeutic axillary treatment are analyzed for their association with the development of BE, with particular emphasis on the fractionation of radiotherapy (RT). Both hypofractionated and normofractionated radiotherapy concepts for breast treatment are examined. For validation, a retrospective cohort of 1156 treatments was available.

## 1. Introduction

Postoperative radiotherapy (RT) is the standard of care after breast-conserving surgery in the curative treatment of breast cancer [1]. RT has a significant impact on local and regional cancer control, with tolerable side effects.

In the future, genetic and post-genetic profiles may determine the therapeutic options; however, RT is currently the standard of care after any breast conservation surgery [2,3].

However, in this context, breast edema (BE) after RT might impact the quality of life of surviving patients [4,5]. The two available internationally used classifications grade post-therapeutic BE as follows:

- “Late effects on normal tissues, in subjective, objective, management and analytic categories “ (LENT-SOMA) differentiates between “asymptomatic—grade 1” and “symptomatic—grade 2,” with medical interventions being graded as “grade 3” and surgical interventions graded as “grade 4” [6]. However, this does not reflect clinical reality, as in everyday language use, “asymptomatic” would mean “the absence of any symptoms—grade 0.” Furthermore, “medical intervention” is not further specified, leading to significant uncertainties in applying this classification.
- The second system is “CTC 2.0”, which provides general definitions [7]. It differentiates between “none/normal—grade 0,” “mild lymphedema—grade 1,” “moderate lymphedema requiring compression—grade 2,” “severe lymphedema limiting function—grade 3,” and “severe lymphedema limiting function with ulceration.”

It is therefore difficult to differentiate reliably between “mild” and “moderate” BE.

These gaps in the international classifications are reflected in the toxicity reporting of the large studies on the fractionation of RT as part of breast-conserving therapy for breast cancer. In most of these studies, only “symptomatic edema” is recorded, without further specification [8].

We introduce a clinically meaningful classification of post-RT BE to close this gap. It focuses on the clinical consequences and therapeutic interventions.

## 2. Materials and Methods

The present study involves a retrospective evaluation of the clinical routine at our RT center in Westerstede, Lower Saxony, Germany. The data were collected exclusively from files maintained at our institution. As established during the admission process in our department, only patients who provided consent allowing their data to be used for anonymized retrospective analysis were included. The data processing and evaluation adhered to the guidelines of the Ethics Committee of Hanover Medical School, Germany, No. 10516_BO_K_2022.

We analyzed all patients who received postoperative RT of the entire breast in a breast-conserving therapy with curative intention between 2011 and 2021.

The RT planning adhered to internal standards and aimed to meet the ICRU criteria. Quality assurance was maintained as a daily routine, like the “4 eyes principle” in target volume delineation and physical RT planning. During RT, the patients were visited once weekly by a radiation oncologist. After RT, the patients were examined after 3, 12, 36, and 60 months, in case of clinical problems and therapy-related toxicity, with more frequent follow-ups as required. These examinations included an anamnesis and—among others—the inspection and palpation of both breasts. All findings were documented and graded in a standardized form according to LENT-SOMA, the CTC, and the system given below.

### 2.1. Data Collection

Our internal patient documentation recorded anamnestic, lifestyle, clinical, and RT planning parameters and toxicity. Data on surgery, pathology, and other therapeutic interventions, such as hormonal therapy, chemotherapy (CTX), or antibody-directed therapies, were extracted from the documentation of our cooperating certified breast cancer center.

### 2.2. Westerstede (WST) Breast Edema Classification

To grade RT-associated BE, we developed a classification system that is not based on LENT-SOMA or CTC 2.0. The grading system comprises four severity levels. The degree of severity is determined by the BE’s therapeutic intervention (Table 1). A severity level is assigned once one of the respective criteria are met.

### 2.3. Statistical Analysis

Differences in quantitative parameters were examined using the Wilcoxon test. For comparing frequencies between groups, the chi-squared test or Fisher’s exact test was applied: The chi-squared test was used when all expected cell frequencies were at least 5. If any expected frequency was below 5, Fisher’s exact test was employed instead. We calculated the hazard ratio (analogous to the Mantel–Haenszel method) for the individual risk factors of the measured collective compared to the unexposed individuals of the total collective. Then, the hazard ratio for the risk-exposed patients in the normofractionated group (nfRT) versus the risk-exposed patients in the hypofractionated group (hfRT) was calculated.

A difference determined by the Wilcoxon test or the hazard ratio calculated by the Mantel–Haenszel method was considered statistically significant at a *p*-value of *p* < 0.05. The results were accepted if they fell within the 95% confidence interval. Statistical analysis was performed using Prism^®^ 10 for Mac (Version 8.00, GraphPad Software Inc., San Diego, CA, USA).

## 3. Results

**Figure 1:**
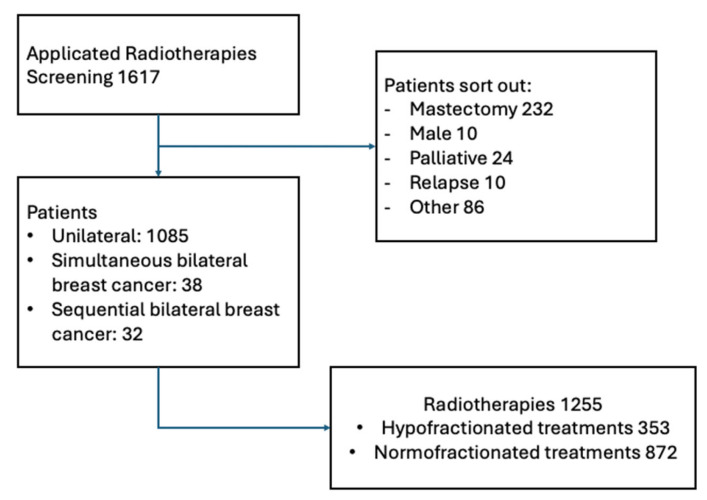
Screening of patients.

We identified 1617 patients with breast carcinoma who received RT as part of their therapy. Those older than 18 years, after breast-conserving surgery, adjuvant RT, and a sufficient follow-up, were included. The exclusion criteria were local therapy alone, male gender, and recurrence therapy with previous RT or refusing the use of data for retrospective investigations. Available for the analysis were 1156 patients who underwent postoperative RT following breast-conserving surgery (Figure 1). Of these, 1085 patients were treated with unilateral breast cancer, 38 patients were diagnosed with simultaneous bilateral breast cancer, and 33 patients developed bilateral breast cancer sequentially.

A total of 872 patients received nfRT, while 353 received hfRT. Detailed patient characteristics are given in Table 2.

Comparing the two groups, the hfRT group was older (8.388 ± 0.7030 years, *p* < 0.05), and vascular comorbidities were twice as prevalent. However, the nfRT group comprised three times more active smokers (*p* < 0.05) and more patients suffering from hypertension (*p* < 0.05). In the hfRT group, more patients had smaller primary tumors without nodal involvement. This was reflected in the applied treatments, with significantly more sentinel biopsies as the only axillary intervention (*p* < 0.05) and less CTX (*p* < 0.05). Furthermore, in the hfRT group, only 5% of patients received RT of the lymphatics, whereas all other patients received treatment only for the breast (*p* < 0.05). In the nfRT group, more than 80% received an additional boost dose to the tumor bed, while in the hfRT group, only 1/3 received such a dose escalation (*p* < 0.05).

During follow-up, 407 BEs (33% of the collective) were reported according to the WST classification, compared to 213 according to the CTC (17%).

Thus, applying the WST criteria resulted in a significantly higher incidence of BE (chi-squared test: *p* < 0.0001) (Table 3).

However, the CTC resulted in more grade II BE than grade I, in contrast to the WST classification.

Between hfRT and nfRT, there was no difference in the chi-squared test for the incidence of grade I and II BE, irrespective of the applied classification. According to any classification, grade III BE was not recorded in the entire collective (Table 3).

The clinical course of the diagnosed and treated BE was favorable (Table 3): in approximately 70% of the affected patients, complete remission was achieved during further follow-up care; however, in 16% of the affected collective, the clinical course remained unknown due to loss to follow-up or severe competing morbidities.

We analyzed potential risk factors for the development of post-RT BE, defined as “at least grade 1 according to the WST classification”, in the entire collective (Table 4).

**Figure 2:**
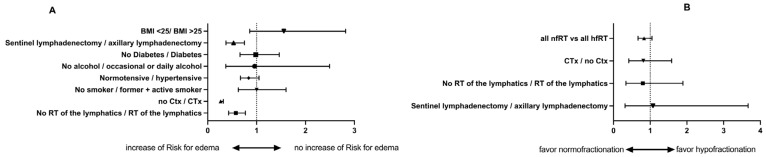
Univariate model: (**A**) The influence of potential risk factors, and (**B**) the confirmed risk factors from the univariate model and the impact of fractionation. For the confirmed risk factors, the fractionation has no influence on the risk of edema.

A significant hazard ratio for the development of BE was determined for the RT of the lymphatic drainage pathways (*p* = 0.00002), the application of CTX (*p* < 0.0001), and complete axillary dissection compared to sentinel node dissection (*p* = 0.0004) (see Figure 2A). No influence on the BE risk was seen for AHT, smoking habits, hypertension, alcohol intake, or diabetes.

The hazard ratio for developing BE was not significantly higher in the hfRT group (0.8337, 95% CI 0.6688–1.049, *p* = 0.1219) according to the Mantel–Haenszel calculation. All potential risk factors concerning RT fractionation were additionally analyzed (see the bottom half of Table 4: **nfRT vs. hfRT).** Under none of the identified risk factors did the choice of fractionation exert an additional negative impact on the risk of developing BE—an increased risk of edema could not be identified for any risk factor after hfRT compared to nfRT. Thus, fractionation of RT did not show any influence on the development of BE in the three factors significantly influencing the outcome (axillary dissection, CTX, and RT of the lymphatics; see Figure 2B).

Additionally, the risk of grade II BE was not higher after hfRT compared to nfRT (1.036, 95% CI 0.6511–1.650, *p* = 0.8805).

The multivariate analyses confirmed that the RT of the lymphatic drainage pathways (*p* = 0.031) and complete axillary dissection compared to sentinel node dissection (*p* = 0.044) were significant risk factors for BE. However, in this analysis, CTX was not confirmed as a risk factor for BE (*p* > 0.05). Additionally, other influencing factors, such as BMI, diabetes, alcohol consumption, hypertension, and smoking habits, did not significantly influence the BE risk in the multivariate model (all *p* > 0.05).

The ROC model was statistically significant in describing the association between axillary lymph node dissection and lymphatic pathway irradiation and the increased probability of developing BE (*p* < 0.0001). The model yielded an area under the curve (AUC) of 0.58, with a 95% confidence interval ranging from 0.543 to 0.615, indicating a modest discriminatory ability (additional ROC curve (Figure S1) and multivariant Plot (Table S1) available in the Supplementary Materials).

## 4. Discussion

The data presented in this study show no significant difference in the incidence of BE between patients treated with nfRT and hfRt after breast-conserving surgery.

The results are thus consistent with the prospective randomized studies investigating hfRT.

The Start-Trial showed no significant difference between the standard 50 Gy arm and 41.6 Gy hfRT arm A [9]. The risk of edema was even reduced in the hfRT group of trial arm B, with a dose of 39 Gy. This implies a dose-dependent risk of developing BE.

These findings of a dose-dependency of post-RT BE are supported by the toxicity profiles of the IMPORT HIGH trial, in which the risk of BE was reduced in the experimental groups. Experimental arm 1 received 36 Gy in 15 fractions to the whole breast, 40 Gy in 15 fractions to the partial breast, and a simultaneous boost of 48 Gy in 15 fractions to the tumor bed. In contrast, experimental arm 2 received an escalated dose of 53 Gy to the tumor bed [10]. After 5 years, a reduced incidence of BE was observed, with rates of 5.2% in arm A and 5.7% in arm B, compared to 8.6% in the standard arm (40 Gy in 15 fractions, followed by a sequential boost of 16 Gy in 8 fractions).

Reducing RT toxicity, such as the BE risk, is particularly important in multimodal therapy for low-risk tumors in elderly patients [11,12]. A risk-adapted RT with partial de-escalation [13] through graduated dose de-escalation (IMPORT HIGH [10]) or partial breast RT (IMPORT LOW [14]) seems to be better tolerated, with fewer effects on quality of life than adjuvant hormonal therapy [15].

However, standardized criteria for recording BE after RT are lacking. In our cohort, we reported rates of 35%/19% in the nfRT group and 28%/14% in the hfRT group according to WST/CTC, respectively. In comparison, no more than 9% of patients were affected by radiogenic BE in the START trials [9,16], and only about 2% in the HYPO study [8]. These vast differences can be explained by the different ways of assessing and categorizing BE [5].

The START trials utilized clinical assessment and scored using the contralateral breast as a comparison, along with a four-point graded scale [16], which was previously introduced in a pilot study [17]. In the HYPO study, however, BE was only recorded from at least grade II, with corresponding symptoms according to the LENT-SOMA scale [8,18]. Thus, most post-RT BEs are classified as asymptomatic [19]. When standardized scoring systems for side effects after RT are compared, differences in the incidence of side effects are observed depending on the measurement method [20]. Imaging-supported scoring systems for an objective evaluation, like for the fat grafting in the aesthetic setup [21], are not available. However, as the density of the breast also influences the RT dose distribution, this must also be taken into account by the chosen imaging modality [22].

Our WST scoring system categorizes BE based on the therapeutic consequences required for its treatment. As a result, we recorded a higher number of BE cases that would have otherwise remained subclinical according to the CTC or LENT-SOMA. However, the CTC classification resulted in a relatively high incidence of grade II BE. This can be explained by the imprecise definition of “need for compression”, ranging from the use of compression wear to the need for professional lymphatic drainage. The WST classification resulted in a larger incidence of grade I BE cases, which were managed with self-applied basic treatment, such as auto-drainage.

In about 70%, a regression of the BE was achieved in the clinical course, or at least a regression to a level at which physiological drainage was sufficient for adequate symptom control.

Another problem arising from the inconsistent definition of BE in the studies is the characterization of risk factors in the context of breast RT including the lymphatics. In large hypofractionation studies on prostate cancer, like the HYPRO-Trial, both treatment-related and patient-related risk factors for the development of early and late toxicity in the subgroups of the characteristics were examined in detail [23,24]. For breast cancer, comparable analyses for subgroups are insufficiently available in the large hypofractionation studies and are entirely missing in AMAROS [25] and SENOMAC [26].

A 2019 publication by Young-Afat et al. contains subgroup analyses of treatment-related risk factors [4]. Here, systemic therapy and ALND increased the risk of BE; however, neither AHT nor SNLE had any influence. Our analysis of the treatment-related risk factors in the univariate model confirms these results. Furthermore, there was no evidence in our collective that hypofractionation contributes to an increased risk of BE formation in the presence of treatment-associated risk factors. It could also be discussed whether different surgical techniques of ALND have an influence on the risk of BE [27].

We did not identify any patient-related risk factors that increased the risk of BE after RT. Postoperative RT and smoking history are associated with late complications for head and neck cancer patients [28]. This might be the case for breast cancer patients as well. However, the literature regarding smoking as a patient-related risk factor is ambivalent [28,29,30]. Furthermore, when collecting data on smoking, there is a difficulty in clearly quantifying the extent of consumption. In our retrospective study, only information on smoking behavior was available from the medical history. An estimate of consumption in pack-year units was only available in a minority of cases. We do not want to exclude negative influences of smoking on BE with our data, but we cannot further characterize it at this point.

In general, our study has limitations. Although the RT planning, examination, and classification of the patients were very homogeneous, the retrospective analysis led to an unequal distribution of most risk factors in the two fractionation groups. This ultimately reflects the gradual introduction of hypofractionation into clinical routine.

## 5. Conclusions

The WST BE classification allows us to characterize post-RT BE by aligning it with treatment management. This results in recording BE cases that would otherwise remain undetected as “subclinical” according to other classifications.

Fractionation of RT did not influence the BE risk. Other treatment-related risk factors described in the literature, such as RT of the lymphatic drainage pathways or the extent of axillary dissection, were confirmed in the analysis.

## Figures and Tables

**Table 1:** WST breast edema classification in comparison to CTC 2.0.

Grade	0	I	II	III
CTC 2.0	No symptoms	Symptomatic edema	Need for compression	Severe lymphedema limiting function with ulceration
WST	No therapy	Lymphatic drainage performed by the patient	Professional lymphatic drainage	Surgical intervention

**Table 2:** Patient characteristics according to nfRT and hfRT; * *t*-test.

Category	Total n = 1225	nfRT n = 872	hfRT n = 353	Significant Difference?
Mean age	60.4	58.0	66.4	yes (*p* < 0.05)
<40	47 (3.8%)	42 (4.8%)	5 (1.4%)	yes (*p* < 0.05).
40–49	171 (13.9%)	153 (17.55%)	18 (5.1%)	yes (*p* < 0.05).
50–64	539 (44.0%)	419 (48.0%)	120 (34.0%)	yes (*p* < 0.05).
65–74	310 (25.3%)	199 (22.8%)	111 (31.4%)	yes (*p* < 0.05).
>75	158 (12.9%)	59 (6.7%)	99 (28.0%)	yes (*p* < 0.05).
Mean BMI	27.09	27.51	26.08	yes (*p*< 0.05) *
BMI > 25	751 (61.3%)	541 (62%)	210 (59%)	no (*p* = 0.44)
Left/right	640/585	462/410	178/175	no (*p* = 0.45)
Risk factors				
Active smokers	151 (12.3%)	137 (15.7%)	14 (4.0%)	yes (*p* < 0.05).
Former smokers	97 (7.9%)	60 (6.9%)	37 (10.5%)	yes (*p* < 0.05).
Alcohol consumption				
- Never	614 (50.1%)	436 (50%)	178 (50.42%)	no (*p* = 0.94)
- Occasionally	552 (45.0%)	388 (44.5%)	164 (46.5%)	no (*p* = 0.57)
- Daily	44 (3.6%)	37 (4.2%)	7 (2%)	yes (*p* < 0.05).
Hypertension	693 (56.6%)	532 (61.0%)	161 (45.6%)	yes (*p* < 0.05).
Vascular diseases	77 (6.29%)	42 (4.82%)	35 (9.9%)	yes (*p* < 0.05)
Non-insulin-dependent diabetes	83 (6.8%)	59 (6.8%)	24 (6.8%)	no (*p* = 1)
Insulin-dependent diabetes	21 (1.7%)	16 (1.8%)	5 (1.4%)	no (*p* = 0.80)
T-status				
pTis + Tmi/ypT0 + ypTis + ypTmi	14 (1.1%)/90 (7.3%)	12 (1.38%)/73 (8%)	2 (0.5%)/17 (5%)	yes (*p* < 0.05).
pT1/ypT1	739 (60.3%)/61 (4.9%)	484 (56%)/50 (6%)	255 (72%)/11 (3%)	yes (*p* < 0.05).
pT2/ypT2	258 (21.0%)/24 (1.9%)	199 (23%)/22 (3%)	59 (17%)/2 (0.6%)	yes (*p* < 0.05).
pT3/ypT3	27 (2.2%)/4 (0.3%)	22 (3%)/4 (0.5%)	5 (1%)/0	no (*p* = 0.15)
pT4/ypT4	7 (0.6%)/1 (0.08%)	5 (0.6%)/1 (0.1%)	2 (0.6%)/0	no (*p* > 0.99)
N-Status				
pN0,pNmi/ypN0	832 (67.9%)/144 (11.7%)	537 (62%)/116 (13%)	295 (84%)/28 (8%)	yes (*p* < 0.05).
pN1/ypN1	176 (1.4%)/25 (2.0%)	151 (17%)/23 (3%)	25 (7%)/2 (0.6%)	yes (*p* < 0.05).
pN2/ypN2	24 (1.9%)/9 (0.7%)	22 (3%)/9 (1%)	2 (0.6%)/0	yes (*p* < 0.05).
pN3/ypN3	13 (1%)/2 (0.2%)	12 (1%)/2 (0.2%)	1 (0.3%)/0	yes (*p* < 0.05).
Primary CTX	182 (14.9%)	150 (17.2%)	32 (9.0%)	yes (*p* < 0.05).
Adjuvant CTX	316 (25.8%)	269 (30.9%)	47 (13.3%)	yes (*p* < 0.05).
AHT	1037 (84.7%)	728 (83.5%)	309 (87.5%)	no (*p* = 0.09)
Sentinel lymphatic biopsy	1061 (86.6%)	722 (82.8%)	339 (96.0%)	yes (*p* < 0.05).
Axillary dissection	146 (11.9%)	137 (15.7%)	9 (2.6%)	yes (*p* < 0.05).
RT target volumes				
Breast only	959 (78.3%)	627 (71.9%)	332 (94.0%)	yes (*p* < 0.05).
Lymphatics	216 (17.6%)	198 (22.7%)	18 (5.1%)	yes (*p* < 0.05).
- Supraclavicular	105 (8.6%)	100 (11.5%)	5 (1.4%)	yes (*p* < 0.05).
- Axillary	18 (1.5%)	12 (1.4%)	6 (1.7%)	no (*p* = 0.61)
- Axillary + supraclavicular after positive sentinel	76 (6.2%)	69 (7.9%)	7 (2%)	yes (*p* < 0.05).
- Parasternal	17 (1.4%)	17 (1.9%)	0 (0.0%)	yes (*p* < 0.05).
Simultaneous boost (tumor bed)	744 (60.73%)	622 (71.3%)	122 (34.6%)	yes (*p* < 0.05).
Sequential boost (tumor bed)	102 (8.3%)	97 (11.1%)	5 (1.4%)	yes (*p* < 0.05).

**Table 3:** Characteristics of BE and therapeutic consequences according to the WST and CTC classifications.

	All (n = 1225)	nfRT (n = 872)	hfRT (n = 353)	Significant Difference?
**WST classification**				
All	407 (33%)	308 (35%)	99 (28%)	yes (*p* < 0.05)
I°	298 (24%)	225 (26%)	73 (21%)	yes (*p* < 0.05)
II°	109 (9%)	83 (9%)	26 (7%)	no (*p* = 0.268)
**CTC classification**				
All	213 (17%)	162 (19%)	51 (14%)	no (*p* = 0.07)
I°	82 (7%)	61 (7%)	20 (6%)	no (*p* = 0.45)
II°	131 (10%)	101 (12%)	31 (9%)	no (*p* = 0.19)
**Clinical course of BE (according to WST)**	407	308	99	
Complete remission	281 (69%)	218 (70%)	63 (64%)	no (*p* = 0.21)
Persistent grade I on the last examination	39 (9%)	31 (10%)	8 (8%)	no (*p* = 0.70)
Persistent grade II on the last examination	23 (6%)	17 (6%)	6 (6%)	no (*p* = 0.80)
Unknown	64 (16%)	42 (14%)	22 (22%)	Not applicable

**Table 4:** Hazard ratios for BE risk (WST classification) according to the univariate model for the entire collective and risk factors compared between nfRT and hfRT, with a significance level of *p* < 0.05.

All	Hazard	95-%	Significant?	*p* Value
No RT of the lymphatics/RT of the lymphatics	0.5744	0.4286–0.7698	yes	0.0002
No hormonal therapy/hormonal therapy	1.010	0.7402–1.378	no	0.9511
No CTX/CTX	0.263	0.263–0.3191	yes	>0.0001
Non-smoker/former + active smoker	1	0.6245–1.602	no	0.9997
Normotensive/hypertensive	0.8374	0.6688–1.049	no	0.1219
No alcohol/occasional or daily alcohol	0.9594	0.3695–2.491	no	0.9322
No Diabetes/diabetes	0.9832	0.6596–1.463	no	0.9335
Sentinel lymphadenectomy/axillary lymphadenectomy	0.5288	0.3727–0.7505	yes	0.0004
**nfRT vs. hfRT**				
No RT of the lymphatics/RT of the lymphatics	0.8006	0.3386–1.893	no	0.6126
No hormonal therapy/hormonal therapy	1.166	0.8901–1.527	no	0.2648
No CTX/CTX	0.8138	0.4176–1.586	no	0.5448
Non-smoker/former + active smoker	1.892	0.4781–6.191	no	0.2919
Normotensive/hypertensive	1.327	0.0917–1.926	no	0.1374
No alcohol/occasional or daily alcohol	1.327	0.9137–1.926	no	0.1374
No Diabetes/diabetes	1.327	0.9137–2.195	no	0.1340
Sentinel lymphadenectomy/axillary lymphadenectomy	0.8535	0.3319–3.668	no	0.7224

## Data Availability

The data presented in this study are available on request from the corresponding author.

## Supplementary Materials

The following supporting information can be downloaded at https://www.mdpi.com/article/10.3390/cancers17142368/s1: Figure S1: ROC-model; Table S1: multivariant Plot.

## Abbreviations

The following abbreviations are used in this manuscript:

AHT	Anti-hormonal therapy
BE	Breast edema
BMI	Body mass index
CTC	Common Toxicity Criteria
LENT-SOMA	Late effects on normal tissues, in subjective, objective, management and analytic categories
WST	Westerstede
CTX	Chemotherapy
RT	Radiotherapy
hfRT	Hypofractionated radiotherapy
nfRT	Normofractionated radiotherapy
ALND	Axillary lymph node dissection
SNLD	Sentinel node biopsy
