# Supplementary material for: Breast Edema After Breast-Conserving Surgery and Radiotherapy: Introduction of a Clinically Meaningful Classification and Evaluation of the Incidence After Normo- and Hypofractionated Treatments
Source: Cancers (Basel). 2025 Jul 16;17(14):2368. doi: 10.3390/cancers17142368 (PMC12293687; doi:10.3390/cancers17142368)
Supplement: Supplementary file 1 [file cancers-17-02368-s001.zip › cancers-3711457-supplementary.pdf]

**Table S1.** Multivariant Plot. Details of the multivariant analysis.

| Model | | | | | *p* |
|---|---|---|---|---|---|
| **Parameter estimates** | **Variable** | **Estimate** | **Standard error** | **95% Cl** | |
| β0 | Intercept | -0,7599 | 0,2018 | -1,161 to -0,3688 | <0.05 |
| β1 | Rauchen | -0,1126 | 0,09537 | -0,3032 to 0,07140 | 0.2377 |
| β2 | Alkohol | 0,07631 | 0,1255 | -0,1701 to 0,3221 | 0.5431 |
| β3 | art, Hypert, | 0,1436 | 0,1350 | -0,1216 to 0,4078 | 0.2874 |
| β4 | DM | -0,2295 | 0,1915 | -0,6182 to 0,1361 | 0.2874 |
| β5 | CTx | -0,1870 | 0,1516 | -0,4874 to 0,1074 | 0.2307 |
| β6 | HTX | -0,08061 | 0,1863 | -0,4419 to 0,2895 | 0.2173 |
| β7 | SLNB/ALND | 0,5097 | 0,2364 | 0,04496 to 0,9733 | 0,0310 |
| β8 | Rtx-LAW | 0,4073 | 0,2023 | 0,007340 to 0,8021 | 0.0440 |

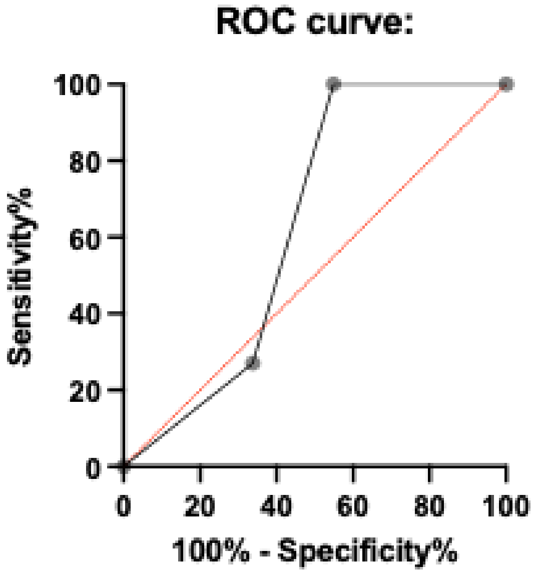


**Figure S1.** ROC-model.
